# Addressing mental health care needs of vulnerable migrants by public psychiatry in France: the CAPSYS clinic

**DOI:** 10.3389/fpsyt.2026.1773564

**Published:** 2026-07-30

**Authors:** Andrea Tortelli

**Affiliations:** 1 Pôle Psychiatrie Précarité, Groupe Hospitalier Universitaire Paris psychiatrie & neurosciences, Paris, France; 2 ESSMA, Institut Pierre Louis d’Epidemiologie et de Sante Publique, Paris, France; 3 Health Department, Institut Convergences Migrations, Paris, France

**Keywords:** integrated care, mental health, mental health provision, refugee and asylum seekers, vulnerable migrants

## Abstract

Forced migration and post-migration stress are leading to poor mental health among immigrants in Europe, placing considerable strain on mental healthcare systems. In France, in light of these challenges, the Regional Health Agency in Greater Paris took the initiative to fund the first public psychiatric consultation for migrants, CAPSYS. Methods: The present article will discuss the establishment of the clinic and the characteristics of the population it serves. Results: Since 2021, CAPSYS has received about 2000 patients, mostly men, coming from Afghanistan and sub-Saharan Africa. Post-traumatic stress disorder, combined with symptoms of anxiety and depression, was present in one patient in two. Constant adaptations were required to facilitate access to care in the context of restrictive immigration policies.

**Conclusion:**

Mental health care provision for vulnerable migrants in France has become a public health issue. Social determinants of health and ensuring access to care must be considered to prevent and treat mental health problems.

## Introduction

The recent wave of migration to Europe, the largest since the Second World War, is putting mental healthcare systems to the test, due to a population particularly exposed to multiple risk factors for the development of mental health problems, before, during, and after migration (1–3).

In France, applications have tripled since 2015, reaching more than 100,000/year, mainly from Afghans, followed by sub-Saharan Africans. France can be the first choice for French-speaking migrants from former colonies, but, as in other European countries, most migrants’ choice is driven by practical considerations, particularly following rejections from other countries or a perception of better prospects for success or a warmer welcome. The procedure is lengthy, and access to accommodation, benefits, and employment is limited. At the end, seven out of ten asylum seekers are refused, leading to an irregular situation and homelessness in most cases (4–6). These facts have been associated with the worsening of their physical and mental health, as well as with barriers to access to care, becoming one main concern of professionals working with this population (7–9). Data from the Greater Paris shows that the most outreached are those presenting common mental health disorders and experiencing unstable housing (10). Both non-governmental organizations (NGOs) and public services are overrun, leading to referrals that are based on geographical distance, appointment, and interpreter availability rather than on the complexity or severity level of mental healthcare needs (11).

This situation is largely explained by a widespread belief in France regarding the need for specific knowledge to provide care to this population, theorised by ethnopsychiatry and psychoanalysis, delegating first-line mental health care provision to NGOs, despite unconditional mental health care provision by public psychiatry (12–14). Nevertheless, NGOs primarily offer counselling and psychotherapy services, possess limited capacity, and are predominantly located in urban areas. In contrast, the public psychiatric system is focused mainly on managing severe and chronic psychiatric disorders, such as psychosis, and often underestimates the impact of social determinants of health and post-migration stress on their development and outcome (15–17).

Public mental health services and NGOs models in France are then failing to provide efficient care for this population, calling for an integrative approach at the intersection of psychiatric, somatic, and social needs, as largely recommended in recent years (18–20).

## Aims and methods

This community-based case study aims to describe the CAPSYS clinic, including its conceptual framework, implementation context, missions, organisation, and day-to-day operations. The model’s strengths and limitations, as well as its reproducibility and transferability, are also discussed.

### Analysis

Descriptive data on service users’ sociodemographic characteristics and clinical aspects are presented as percentages. Comparison of findings will be made with the literature.

## Results

### Context

CAPSYS (Consultation et Accompagnement Psychiatrique et Social - Psychiatric consultation and social coordination) is the first public psychiatric clinic in France dedicated to vulnerable migrants, such as asylum seekers and migrants living in precarious situations. The multidisciplinary team comprising psychiatrists, nurses, social workers, psychologists, and mediator-interpreters receives referrals from medico-social services in Greater Paris, including emergency and health services, outreach teams, NGOs, or any other professional in contact with this population. CAPSYS has been permanently funded by the Ile-de-France Regional Health Agency (ARS-Ile de France) since 2021, and is part of the French public psychiatry system, represented in Paris by the Groupe Hospitalier Psychiatrie Neurosciences Paris (GHU-Paris).

### Theoretical framework

The clinic’s structure and operational model were designed to address the barriers to care and the specific needs of this population. European studies have identified the main obstacles faced by newly arrived migrants as limited entitlements, language barriers, unmet social support needs, low health literacy, insufficient cultural responsiveness, and discrimination (18, 21–24). On the other hand, evidence suggests that organisational flexibility, interdisciplinary collaboration, and meaningful patient involvement improve the identification of needs and the coordination of follow-up, with measurable benefits for the quality of life of vulnerable groups (25–28).

In France, we observed similar barriers and needs (10, 11). The project also aims to respond to local priorities identified on the Health Ministry’s Regional Mental Health Project: 1) early detection of mental disorders; 2) access to diagnosis, care and support in accordance with the latest scientific data and good professional practice; 3) access for people with mental disorders to physical care tailored to their needs; 4) a seamless, high-quality health and life style pathway; 5) action on the social, environmental, and territorial determinants of mental health; 6) prevention and management of crisis and emergencies (29).

### Eligibility

Patients over the age of 15 (criteria for adult psychiatry in France) who require outpatient psychiatric care for common mental disorders. On the other hand, the clinic is not supposed to be a source of inequality of access to care; therefore, it is not a substitute for emergency or inpatient care, nor for follow-up for severe to long-term disorders such as psychosis or disabilities requiring comprehensive care by other facilities from the psychiatric public system, such as a day hospital or adapted housing. In the same line, patients for whom addiction is the main/first-line diagnosis are not admitted to the clinic for reasons relating to the specific facilities required for addiction care provision in France.

### Functioning

In practice, throughout the follow-up process, medico-social priorities are identified with the patient to establish procedures and referrals (e.g., social rights, accommodation, language learning). This patient-centred care involves cultural humility by respecting and responding to patients’ individual preferences, needs, and values, integrating them into all clinical decisions (30, 31). A walk-in service has been established to address the challenges faced by individuals experiencing unstable housing and forced mobility, to promote continuity of care. Psychiatric follow-up and overall coordination of the situation are effective for as long as it takes to establish a referral adapted to the clinical picture and living conditions, despite the instability of the living environment. This makes it easier to see and understand how the mental health partners interact with the patient, giving them greater decision-making power and ensuring that their priorities are taken into account.

Somatic co-morbidities are common in this population, particularly due to exposure to violence and infectious diseases before, during, or after migration (32–34). Additionally, chronic pain, somatization, and the psychological consequences of serious physical illnesses are intertwined with the development and outcome of psychiatric disorders (35–37). In France, access to healthcare is a right guaranteed, including for those in an irregular situation (38). However, healthcare coordination is necessary for complex cases due to frequent health illiteracy and the need for interpreters. Helping people to manage stress and strengthening protective and resilience factors, such as social capital, are also part of a prevention strategy, by the provision of psychosocial support and promotion of social capital through cultural activities and partnerships.

Finally, in addition to the fundamental need to evaluate the clinic’s operations and care, a research program is established to continue investigating risk and resilience factors, as well as clinical issues related to mental health problems in this population. This knowledge, together with that gained from day-to-day practice, is disseminated through training courses and scientific communications.

### Cultural issues

Recognising cultural differences enables better adherence and outcomes, and reduces diagnostic bias (39–42). One key factor in achieving this is the use of interpreters (43, 44). In the clinic, about 60% of patients speak Dari and/or Pashto. Interpreters from the team, fluent in these languages, facilitate welcome, referrals to medical and social partners, and psychosocial support. They also organise cultural activities and participate in research projects. Interpreting services for other languages are available in person through a partnership between GHU-Paris and interpreting agencies.

### Treatment

Although psychotherapy is more prominent than pharmacological treatments for post-traumatic stress disorder (PTSD) and symptoms of anxiety/depression in this population, the latter are effective and seem better adapted to the living conditions of those served by the clinic (45–47). Pharmacological treatment, in conjunction with psycho/pharmaco-education, requires fewer appointments and provides a prompt response to the most commonly reported symptoms, such as sleep disturbances and anxiety/depression symptoms (48). Access to medication is possible while waiting for social security entitlements to be processed, provided by the GHU-Paris pharmacy. Individual or group brief psychotherapy is employed as a complement or substitute for pharmacotherapy in non-severe cases. Psychosocial support, such as the “Psychological Management Plus” (PM+) program, which is feasible and effective in this population, is delivered by interpreters from the team (49–51).

### Characteristics of the population

**Figure 1:**
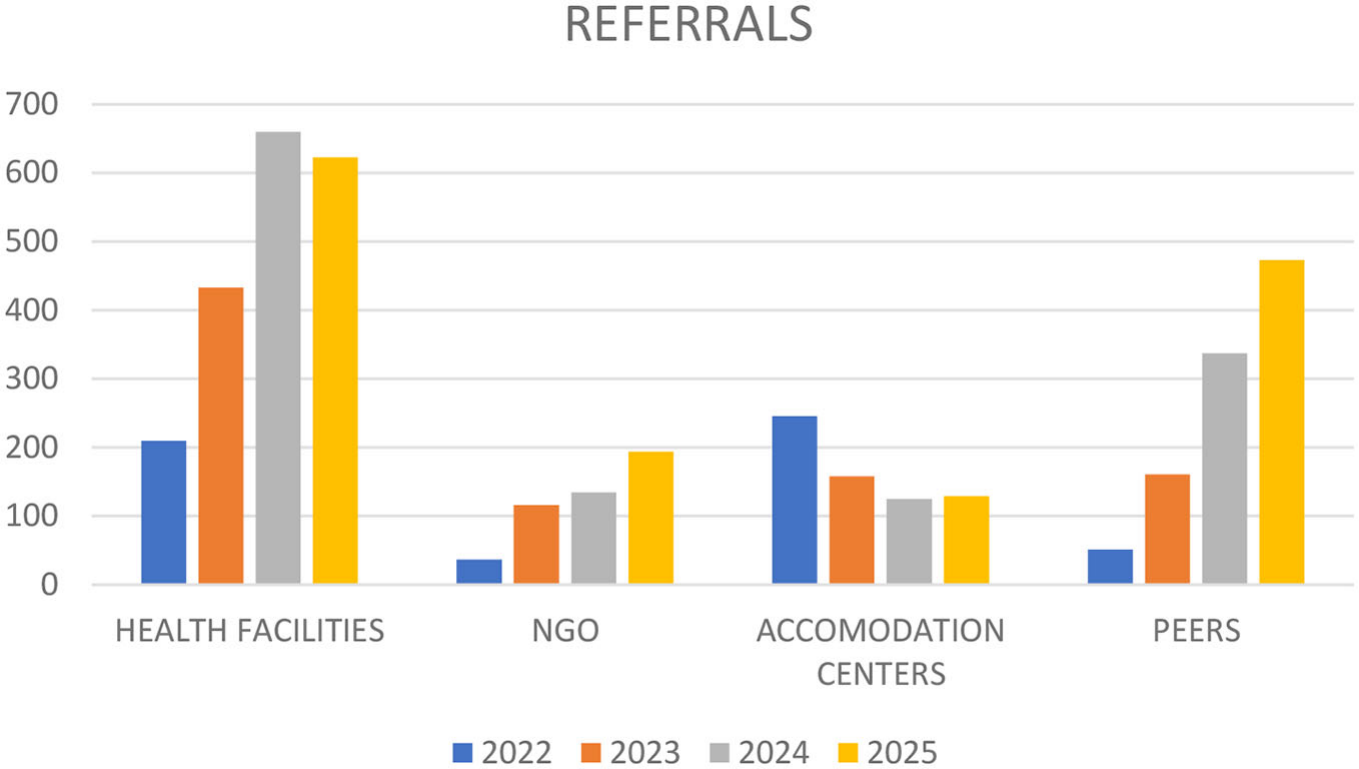
Referrals by year.

Since 2021, CAPSYS has welcomed nearly 2,000 patients with more than 10,000 consultations. The average follow-up period is 18 months, with a turnover mean rate of 58,7% per year, and 78,4% in two years. Socio-demographics and clinical characteristics are shown in **Table 1**. Most patients are Afghan men, non-French speakers, and are seeking asylum. Half of them do not have access to stable housing or economic resources. These proportions have not changed significantly over the years in terms of gender, age, or country of origin. However, an increase in housing instability has been observed. (20% in 2022 to 52,8% in 2025) as well as access to welfare (48, 5% in 2022 to 27,2% in 2025). Referrals are made by healthcare facilities and accommodation or day centers, but currently, one in three patients is referred by word of mouth. This last group has been increasing as a consequence of homelessness, leading to invisibility to outreach teams and social services (**Figure 1**). About one in three patients requires social coordination, and one in five requires somatic coordination. Therapeutic activities (once a month, 15–30 patients) enable patients to break out of isolation and to familiarize themselves with cultural and wellness venues.

**Table 1:** Sociodemographic and clinical characteristics of patients received between 2022 and 2025 (n = 1893).

Origin	Asia	Africa	Other
	61.7%	34.9%	3.4%
Gender (male)	78.3%		
Age	15-24	25-44	> 45
	19.4%	72.5%	8.1%
Married	41.4%		
Family left behind	82.2%		
French speaking	19.8%		
Living conditions	Economic resources	Stable housing	Health insurance
	47.9%*	49.6%	73.4%
Administrative status	Irregular/ Dublin	In procedure	Refugee
	7.2%/5.6%	67.2%	20%
Consultations/year	2-4	5-9	> 10
	35.3%	25.8%	24.9%
ICD-10	F40-49	F30 -39	> 1 diagnosis
	78.6%	14.6%	61%
Pain	Headache	Related to violence	
58,7%	54 %	60 %	
Drug use	Tobacco	Alcohol	Cannabis
23%	63 %	21 %	10 %

*from welfare = 34%.

### Clinical aspects

The most prevalent diagnoses are anxiety, depression, and post-traumatic stress disorders, in line with what is observed in other countries (17, 52, 53). A study conducted between 2022 and 2024 by Melchior and colleagues on a representative sample of 110 patients showed, at the first appointment, a mean score = 21.4 (sd = 7.9) on the Posttraumatic Stress Disorder Checklist (PCL-5), and 31.2 (sd = 11.4) on the Patient Health Questionnaire Anxiety Depression Scale (PHQ-ADS). Psychotic symptoms (mainly auditory hallucinations) were observed in 2% of patients, associated with a PTSD diagnosis. About half of the patients presented moderate to severe PHQ-ADS scores (>18) in addition to probable PTSD (PCL-5 >19) (50, 54, 55).

Clinically, ups and downs are frequent and mostly associated with the asylum procedure and administrative insecurity, as also previously observed in the literature, leading sometimes to despair and suicidal thoughts (56–58). Therefore, the clinic often runs as a crisis facility rather than an outpatient consultation. In this context, walk-in service allows early crisis management with the same team and in the presence of interpreters, avoiding referral to emergency settings and hospitalization in most cases. Since the opening, there have been fewer than ten hospital admissions needed for cases of major depression with catatonia, acute psychosis, or persistent suicidal thoughts.

Finally, straight collaboration with healthcare facilities works in both directions. About one patient in four needs referral: infectious diseases, chronic pain, skin problems, gastrointestinal complaints, and sequelae of physical violence (including excision) are the most common health issues.

### New vulnerable groups identified

There has been an increase in demand in the last months for unaccompanied minors, partly due to the rise in arrivals on French territory, but also because, for most of them, their living conditions are marked by extreme isolation and precarity, which is worsening their mental health, in addition to the negative impact of frequent exposure to violence during the journey (59). The National System for the Protection, Assessment and Guidance of Unaccompanied Foreign Minors estimates that 57% of non-accompanied minors are not recognised as minors (60). For those, access to healthcare and accommodation is hampered; this situation has been recently denounced by the United Nations (61, 62).

Also, acculturation stress, anxiety, and depression symptoms have been observed among wives and children from family reunification following the granting of refugee status to their husbands, mostly from Afghanistan. Difficulties are found to refer the children, underscoring that child psychiatry is even more under-resourced than the adult public sector in France (63).

## Discussion

In this project, we attempt to integrate into public mental health services the main recommendations for effective care provision for vulnerable migrants by considering the social determinants of health, access to services, and the French context. The socio-demographic profile of patients (men from Afghanistan and Sub-Saharan Africa) matches that identified by the Home Office (6), suggesting that the new arrivals are managing to access healthcare, as well as homeless migrants, prevalent in our sample, which is also in accordance with increasing social precariousness due to restrictive immigration policies in France (64). Finally, the clinical profiles, characterized by common mental disorders and PTSD, are the most prevalent observed in this population, which supports the choice of admission criteria (65). To our knowledge, there is no equivalent public-psychiatric service in Europe; a qualitative study on acceptability, as well as a study on cost-effectiveness, are necessary to confirm the model.

### Strengths and limitations

The main lesson learned was that organizational flexibility enabled access to care for individuals not in contact with health or social services, as well as facilitating early crisis management. Also, the importance of network development to ensure the most equitable access to healthcare and social inclusion facilities.

As the first public facility of this kind in France, the main difficulty encountered was the estimation of costs, workforce, and premises, which resulted in the need for a new budgetary plan. This issue took time to resolve, leading to overwork for the team and less efficient reception and care provision. As a result, no-show rates increased because appointments were sometimes given too far in advance, up to six months.

The required competencies of the staff were also misestimated. In addition to cultural competence and knowledge of physical and mental consequences due to forced migration and exposure to violence, an understanding of issues related to precariousness and crisis management was estimated to be necessary. The impact of traumatic events reported by patients on team members, particularly interpreters who have experienced similar situations, has also been considered, and monthly debriefing/supervision sessions with a psychologist have been implemented (66, 67). Finally, barriers to care for children were identified, underscoring the need to also rethink the access to mental health care for these groups at risk (68–70).

### Transferability/reproducibility

There is a large consensus about integrating mental health care into primary care services to facilitate access to vulnerable groups (20, 71–73). However, the French system organisation remains highly fragmented, with integrated care still being at the level of local experiments (74). The discrepancy is explained by the fact that general practitioners are typically self-employed medical doctors, while mental health care provision is mainly provided by the public system. In this context, reproducibility is more feasible than transferability if relying upon straight collaboration with social and health care services. Yet, evaluation of local needs and resources is requested to develop complementary services rather than replicate existing ones, and to build capacity to reassess missions and care provision in line with changing needs. For these reasons, an integrative, community-based approach is preferable to a single theoretical model, as it provides flexibility and maximizes inclusion.

### Is there a need for migrant mental health expertise?

There is no specific treatment for common mental health disorders in migrants. This means that efficient treatment for the observed disorders or symptoms (such as insomnia, anxiety, and nightmares) is largely known and established by scientific literature and is not dependent on migrant status (75–77). In this line, categories of migrants, such as “forced” or “voluntary,” may be misleading in predicting mental health needs: decisions to leave are influenced by the interplay of individual and environmental factors, including political and economic factors, and exposure to trauma may exist during the journey for those who left for economic reasons. Moreover, in the host country, psychosocial risk factors such as poor mental health and barriers to care may negatively affect both categories (78–81). Finally, individual resilience capacity accounts for the development and outcome of mental health problems (82). That said, somatisation may lead to misdiagnosis as an important proxy for anxiety, depression, and PTSD, which can partially explain the high use of somatic or emergency facilities (36, 83). Also, patients’ perceptions of symptoms may be influenced by culture or religion, which could in turn affect help-seeking and coping (84). However, cultural competence training and interventions, which are expected to facilitate access and have a positive impact on outcomes, are rather rare in Europe, and mostly focus on minorities or ethnic status, rather than on individuals’ values (31, 85). Finally, discriminatory attitudes towards migrant patients are observed, resulting in patients being triaged, with those with specific needs or complex situations being excluded (86–89).

Therefore, the main concern is ensuring equitable access to care, driven by immigration and health policies and the practices of healthcare professionals, including the use of interpreters (25, 90).

## Conclusion

There is a growing demand for mental care provision for vulnerable immigrants in France. The CAPSYS provides care through a community-based and integrative approach, considering living conditions and cultural competence. The aim is to have a positive impact on the quality of life and the outcome of mental health problems, as well as effective integration into the mainstream system in the medium and long term. Yet, mental health care provision for this population in France has become a public health issue; large-scale measures, targeting social determinants of health and access to care, must be considered to prevent and treat mental health problems.

## Data Availability

The original contributions presented in the study are included in the article/supplementary material. Further inquiries can be directed to the corresponding author.
